# Experimental and Numerical Investigation in Directed Energy Deposition for Component Repair

**DOI:** 10.3390/ma14061409

**Published:** 2021-03-14

**Authors:** Lan Li, Xinchang Zhang, Frank Liou

**Affiliations:** Department of Mechanical & Aerospace Engineering, Missouri University of Science and Technology, Rolla, MO 65401-0900, USA; ll752@mst.edu (L.L.); liou@mst.edu (F.L.)

**Keywords:** component repair, directed energy deposition, additive manufacturing, damage, deformation, residual stress

## Abstract

Directed energy deposition (DED) has been widely used for component repair. In the repair process, the surface defects are machined to a groove or slot and then refilled. The sidewall inclination angle of the groove geometry has been recognized to have a considerable impact on the mechanical properties of repaired parts. The objective of this work was to investigate the feasibility of repairing various V-shaped defects with both experiments and modeling. At first, the repair volume was defined by scanning the defective zone. Then, the repair volume was sliced to generate the repair toolpath. After that, the DED process was used to deposit Ti6Al4V powder on the damaged plates with two different slot geometries. Mechanical properties of the repaired parts were evaluated by microstructure analysis and tensile test. Testing of the repaired parts showed excellent bonding between the deposits and base materials with the triangular slot repair. 3D finite element analysis (FEA) models based on sequentially coupled thermo-mechanical field analysis were developed to simulate the corresponding repair process. Thermal histories of the substrate on the repair sample were measured to calibrate the 3D coupled thermo-mechanical model. The temperature measurements showed very good verification with the predicted temperature results. After that, the validated model was used to predict the residual stresses and distortions in the parts. Predicted deformation and stress results can guide the evaluation of the repair quality.

## 1. Introduction

Many metallic components are frequently subjected to severe working conditions during service, such as alternating heavy loads, high temperature, high pressure, and wear, which can easily cause defects on these parts. Frequent failures of these components can lead to a severe drop in productivity and process efficiency. Repair or remanufacturing damaged components provides solutions to increase their life economically with minimal interruption in the production process. The typical repair process mainly involves two steps. First, the irregular surface defects are machined by creating a V-shaped groove. Second, suitable materials are deposited back into the damaged zone using welding or additive manufacturing methods [1]. The laser-aided directed energy deposition (DED) process has shown great applications in the field of component repair [2,3]. DED is a typical additive manufacturing process that can create fully dense complex parts by directly melting materials and depositing them on the workpiece layer-by-layer following a user-defined tool path [4,5,6,7]. In this process, a high-power laser with a very small concentration area is used to create a molten pool on the damaged parts. The filler material, usually powders, experiences melting and cooling, and then solidifies to form the deposits. The deposits are usually fully dense and can form an excellent bond with the base parts [8,9] and have high mechanical properties, including tensile strength and fracture toughness [10,11]. For component repair, DED outperforms conventional repair approaches, with the following advantages: (1) precise control of the heating and deposition rate over the geometry and substrate; (2) better compatibility with many advanced materials; and (3) automation capability [12,13,14,15,16].

In the pre-repair process, a V-groove down to the defects is first obtained by removing the defects and surrounding materials so that the DED tools can access the damaged zone. After that, the repair volume is defined, and the damage is then refilled by depositing appropriate materials following the defined repair volume. In the DED process, a high-energy laser beam emits the powder nonuniformly. This high-intensity laser can produce complex thermal history and significant thermal residual stresses. Residual stress induced by a high thermal gradient is likely to cause distortion, cracking, and fatigue failure and impacts the quality of the repaired product. Many endeavors have been made to alleviate the undesirable effects and hence reduce the defects of the finished part. It has been recognized that V-groove geometry imposes a considerable impact on the mechanical performance of finished parts. For example, Graf [17] has analyzed the feasibility of laser metal deposition for refilling different V-groove shapes with both stainless steel and Ti6Al4V by experiments. They reported that the V-groove should be wide enough to ensure a successful rebuild without defects. Pinkerton [18] machined varied V-groove-shaped defects on H13 tool steel substrates and then repaired the missing volumes using the DED process. They concluded that a steep sidewall tilt angle cannot guarantee good metallurgical bonding between the as-deposited material and damaged parts because of the lower laser energy on the steep sidewall. Zhang et. al. [19] repaired three V-shaped defects with varied sidewall inclination angles on H13 tool steel substrates with a cobalt-based alloy. The microstructure and mechanical properties of rebuilt samples revealed that materials can be successfully deposited on H13 tool steel, except for 90° sidewall damage with a lack of fusion and many pores. Some works have tried to accomplish better repair performance under slot geometry with various materials [20,21,22,23]. Paul et. al. [24] reported that they were able to produce fully dense and crack-free WC-Co coatings on low carbon steel with excellent interfacial bonding and much higher hardness. Zhang et al. [25,26,27] repaired damaged compressor blades and damaged dies using the DED process. They concluded that the sidewall inclination angle of the slot should be carefully determined to obtain high-quality repair.

It is instructed that the sidewall tilt angle for V-groove geometry considerably affects the bonding condition of the filler material and substrate since it affects how laser melts the materials on the sidewall. However, there is no direct evidence to demonstrate the causality in the current literature. A clear understanding of that causality helps to optimize the sidewall inclination angle and hence produces good products. Therefore, it is of particular importance to elucidate the fusion conditions and temperature distribution with metallurgical bonding. Previous studies mainly focused on microstructure and performance testing analysis by experiments, finding that it is not easy to measure temperature evolution in the melt pool. In this study, the temperature and stress evolution in the DED process was tracked by both numerical and experimental analysis. With this information, the effect of sidewall inclination angle on the emergence of defects in repaired parts can be clearly understood. The thermal history and laser intensity in the DED process can also be analyzed in order to optimize design or geometry.

The objective of this paper was to investigate the influence of V-groove geometry sidewall angles on the properties of repaired parts through both experiments and numerical modeling. In the experimental part of the study, to perform the repair, V-shaped defects with different sidewall inclination angles were prepared on Ti6Al4V substrates. The repair volume on each substrate was reconstructed by scanning the damaged region using a 3D scanner. After this, the repair toolpath was generated. Then, Ti6Al4V powders were deposited on the damaged parts using the DED process. Subsequently, the repaired parts were tested by microstructure analysis and mechanical testing. In the modeling part of the study, 3D FEA models based on sequentially coupled thermo-mechanical field analysis were developed to simulate the repair process. The simulation accurately predicted temperature and residual stress on the repaired parts. The distortion and residual stress were evaluated to study the influence of the sidewall inclination angles on repair quality. Finally, conclusions were drawn for these two repair samples.

## 2. Experimental Procedure

### 2.1. Material Preparation and Experimental Setup

**Figure 1:**
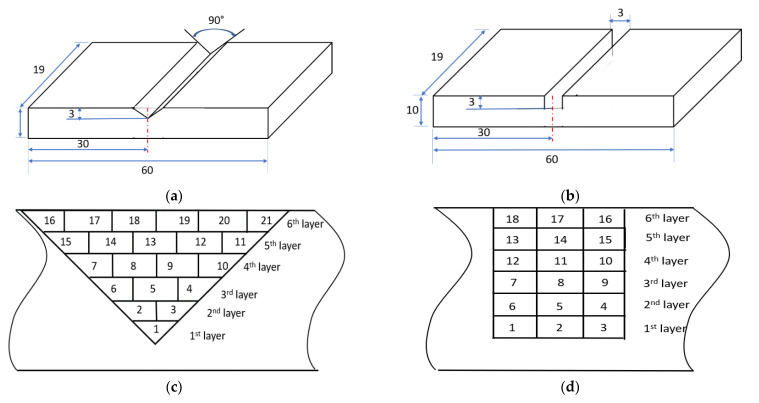
Schematic of the damaged substrates with triangular (**a**) and rectangular (**b**) slots; schematic of the scanning layers and tracks in the damaged substrates with triangular (**c**) and rectangular (**d**) slots.

In order to perform the repair, Ti6Al4V rectangular plates with dimensions of 60 mm × 19 mm ×10 mm were selected as the substrates. Two different slot geometries were machined on the substrates: one triangular with a sidewall inclination angle of 45° (Figure 1a) and one rectangular with a sidewall inclination angle of 90° (Figure 1b). Ti6Al4V powders with particle sizes of 50–150 mm were chosen as the feedstock material. The chemical compositions of Ti6Al4V are listed in Table 1 [28].

The missing volume on each substrate should be determined to obtain the tool path for material deposition. The deposition tool path directly determines the restored geometry and has large effects on the quality of the repaired parts. In order to obtain the missing volume, the algorithm presented in [19] was used. In this process, the missing volume was directly reobtained by scanning the defective area using a structure-light 3D scanner (OptimScan 5M, Shining 3D, San Francisco, CA, USA). After the missing volume was reconstructed, the tool path was generated based on the missing volume. The missing volume was sliced into 6 layers with a layer thickness of 0.5 mm. The tool path consists of an outline contour and a zigzag infill pattern, which indicates that the laser scans the outline first and then the zigzag pattern. The laser tracks for the two different damaged geometries are schematically shown in Figure 1c,d.

After the repair tool path was acquired, the damaged substrates were repaired using a DED system. In this study, the DED system consisted of a YAG fiber laser (IPG Photonics, Oxford, MA, USA) with a peak power of 1 kW, a powder feeding system, a 3-axis worktable, and an enclosure purged with argon gas. During the repair process, the laser beam was kept stationary and the workpiece was moved according to the repair tool path. The processing parameters are listed in Table 2. In the experiment, the substrate was clamped at two ends to prevent rigid body motion. For the experiment planning, the same levels of power, powder flowrate, and traverse speed were used in each experiment.

### 2.2. Sample Preparation and Material Characterization

**Figure 2:**
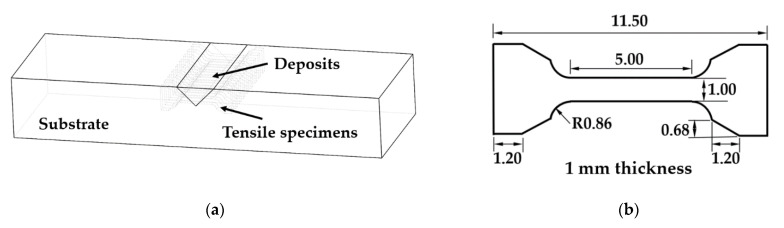
(**a**) Preparation of tensile specimens from repaired parts; (**b**) dimensions of tensile specimens (unit: mm).

The as-deposited samples were sectioned using a Hansvedt (Hansvedt Industries Inc., Rantoul, IL, USA) electric discharge machine (EDM). Then, the samples were ground using silicon carbide abrasive papers from 120 grit to 1200 grit in sequence. Next, the samples were polished using diamond suspensions (9 µm, 6 µm, 3 µm, and 1 µm) and finally polished with 0.04 µm silica suspension. After that, the polished specimens were etched with Kroll’s reagent. The morphology and microstructures were examined using a HIROX KH-8700 (HIROX, Hackensack, NJ, USA) optical microscope and a Helios NanoLab 600 (Thermo Fisher Scientific, Waltham, MA, USA) Scanning Electron Microscope (SEM). The interfacial boundary between the refilled material and substrates was studied. An energy-dispersive X-ray spectroscopy (EDS) line scan was performed from deposits to substrates to analyze elemental composition and distribution. Tensile testing was conducted to test the mechanical properties of the repaired parts. For preparing the tensile specimens, 1.0-mm-thick layers were cut from each repaired substrate, with orientations as illustrated in Figure 2a. Then the tensile specimens were sectioned from each thin slice. The dimensions of the tensile specimens are depicted in Figure 2b. Each tensile specimen consisted of deposits and substrate, with an interface located at the middle of the specimen. The tensile test was conducted using an Instron tester (Model 3300) with a crosshead speed of 0.015 mm/min. Tensile stress-strain curves were obtained.

### 2.3. Microstructure

**Figure 3:**
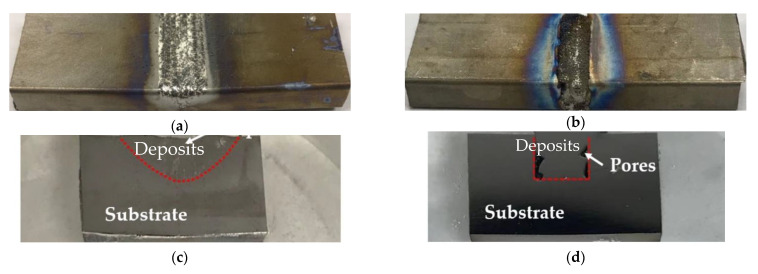
Repaired substrates with triangular (**a**) and rectangular (**b**) slots; overview of the cross-section of the repaired substrates with triangular (**c**) and rectangular (**d**) slots.

**Figure 4:**
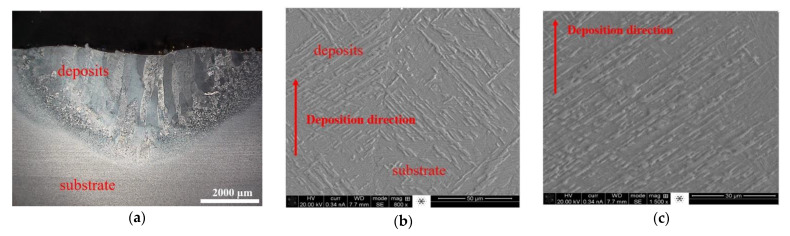

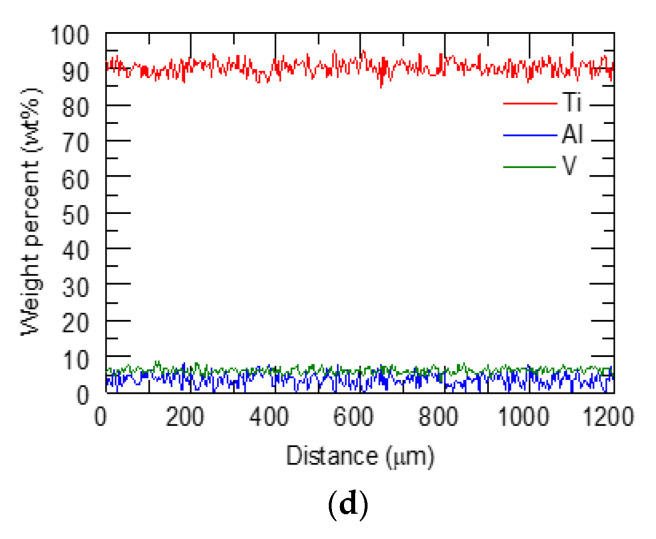
(**a**) Overview of the microstructure of the repaired region; (**b**) magnified view of the interface between deposits and substrate; (**c**) microstructure of deposits; (**d**) elemental distribution over the interface.

Figure 3a,b reveal the images of the repaired parts with triangular and rectangular defects, respectively. An overview of the cross-sections of the triangular and rectangular defects is shown in Figure 3c,d, respectively. It can be observed from Figure 3c,d that the as-deposited materials bonded well with the base part for the triangular case. However, for the rectangular defect case, very large pores were found near the edge of the wall. This is because, in the repair process, the laser cannot access the sidewall due to its vertical geometry. Because of this large pore defect, the following microstructure analysis and tensile test analyses were not performed within the rectangular repair plate. The repaired plate with triangular defects was prepared for microstructure analysis. The micrographs of the cross-sections of the deposits and the interface with the substrate are shown in Figure 4. In Figure 4a it can be seen that the interface between the as-deposited material and the substrate was very clear. In addition, there was no delamination or other defects at the interface, indicating good interfacial bonding. This excellent interfacial bonding was also confirmed by the tensile testing, described in Section 2.4. The deposits were dominated by a columnar microstructure that was growing perpendicular to the interface. These columnar structures were formed due to the directional high thermal gradient along the vertical direction. Micrographs were taken at the deposit–substrate interface (Figure 4b) and of the deposits (Figure 4c), which showed an α+β microstructure, which is typical for additively manufactured Ti6Al4V [29].

EDS line scan analyses were performed across the interface for the triangular case and the result is shown in Figure 4d. The entire transition line spanned approximately 1.2 mm. It is clear that there was no drop or rise in elemental composition over the interface. Additive manufacturing resulted in a uniform distribution of elements across the interface.

### 2.4. Tensile Behavior

**Figure 5:**
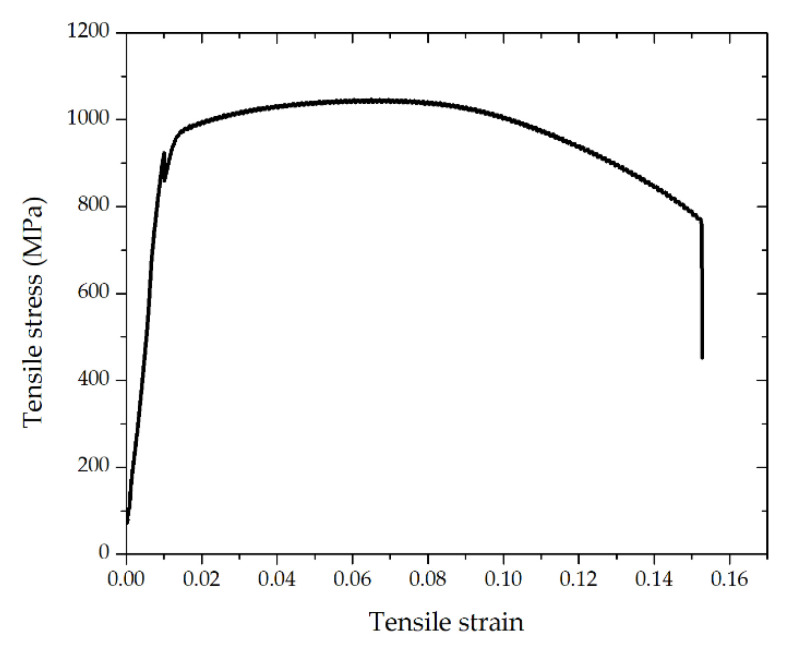
Tensile stress-strain curve.

A total of nine tensile specimens for the triangular repair case were prepared and tested to evaluate the bonding strength between the as-deposited material and the substrate. A representative tensile stress-strain curve is shown in Figure 5. The yield strength and ultimate tensile strength of all the specimens are summarized in Table 3. The testing showed that the yield strength and ultimate tensile strength of the repaired parts were 954 ± 47 MPa and 1037 ± 21 MPa, respectively. More importantly, the samples fractured at the deposit region, not at the interface, indicating a good interfacial bonding strength.

## 3. Numerical Analysis of the Laser Repair Process

### 3.1. Model Setup

In the additive manufacturing process, due to the existence of rapid thermal cycles, residual stress and deformation inevitably occur. Numerical simulation is able to capture the temperature field and thermal stress field in the repair process, which provides a theoretical basis for controlling, adjusting, and reducing residual stress, which has important academic value and practical application significance [30,31,32,33,34,35]. In this section, the Ansys^®^ Mechanical APDL software (Ansys 2020 R1, Ansys, Inc., Canonsburg, PA, USA) was used as the platform to simulate the temperature field and the stress-strain field of the multi-layer laser repair process.

**Figure 6:**
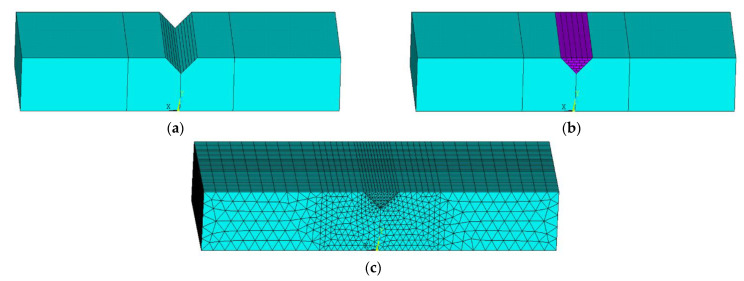
(**a**) FEA model geometry of the damaged part; (**b**) laser scan strategy for repair; (**c**) finite element mesh for directed energy deposition (DED) process simulation.

First, the geometric model with a triangular crack was established to explain the simulation procedure. According to the actual laser deposition process of multi-layer multi-pass, a 6-layer 21-pass geometric model was established. Figure 6a shows the geometry of the 3D numerical modeling domain of the damaged part. The intact section with the damaged slot is shown in Figure 6b,c. Therefore, 21 tracks were planned for the total repair process. The laser transverse speed was 210 mm/min. The element of the new material addition with 0.5 mm thickness was activated sequentially in the laser scanning direction to simulate the material addition process. All processing parameters are listed in Table 4. The domain was meshed with one deposition layer, being one element tall (0.5 mm) and four elements wide. The final model contained a total of 37,920 elements. The finite element mesh configuration of the repair domain is shown in Figure 6c.

### 3.2. Thermal and Mechanical Analysis

The transient temperature distribution in the entire volume of the material can be obtained from the 3D heat conduction equation [36]:

(1) $$\frac{\partial (\rho c_{p}T)}{\partial t}=\frac{\partial }{\partial x}(k\frac{\partial T}{\partial x})+\frac{\partial }{\partial y}(k\frac{\partial T}{\partial y})+\frac{\partial }{\partial z}(k\frac{\partial T}{\partial z})+Q$$

where *k* is the thermal conductivity; *ρ* is the density; *c_p_* is the specific heat; all these thermal-mechanical properties are temperature-dependent; *T* is the current temperature; *Q* is the internal heat generation rate per unit volume; *t* is the time; and *x*, *y*, and *z* are the coordinates in the reference system.

The plate was clamped at the left and right surfaces. Both convection and radiation conditions were considered in all external surfaces, which were applied to all free surfaces. These heat transfer mechanisms are expressed as:

(2) $$\ddot{q}=-k\frac{\partial T}{\partial z}+h\left(T-T_{0}\right)+\varepsilon \sigma \left(T^{4}-T_{0}^{4}\right)$$

where *T* is the temperature of the workpiece, *h* is the heat transfer coefficient of natural convection, which is assumed to be dependent on temperature and is presented in Table 5, *σ* is the Stefan–Boltzmann constant of 5.67 × 10^−8^ W/m^2^∙K^4^ and *ε* is the surface emissivity of 0.3, and T_0_ is the ambient temperature, which is equal to the temperature at the initial time of 25 °C. Moreover, heat conduction at the contact interface between the plate and the clamping system is considered to account for the thermal inertia of the supporting structure. By correlating simulated and experimental results, the heat transfer coefficient used for Newton’s model is set to 100 (W/m^2^·°C).

The stress equilibrium equation is written as [37,38]:

(3) ∇⋅σ=0

where σ is the second-order stress tensor, associated with the material behavior law.

The total strain *ε* component, assuming small deformation thermo-elasto-plasticity, is represented as [39]:

(4) $$\varepsilon =\varepsilon^{e}+\varepsilon^{p}+\varepsilon^{th}$$

where *ε^e^*, *ε^p^*, and *ε^th^* are the elastic strain, plastic strain, and thermal strain, respectively. The isotropic Hooke’s law was used to model the elastic strain (*ε^e^*) in Equation (5). The thermal expansion coefficient was adopted to calculate the thermal strain in Equation (6). Elastic-plastic stain-stress behavior was described by a bilinear stress-strain curve starting at the origin, with positive stress and strain values, which was defined by the elastic modulus *E*, Poisson’s ratio *ν*, yield strength *σ^Y^*, and tangent modulus *G*. This is called the bilinear isotropic hardening (BISO) model in ANSYS software.

The mechanical constitutive law can be written as [39]:

(5) $$\sigma =C\varepsilon^{e}$$

where *C* is the fourth-order material stiffness tensor and *ε^e^* is the second-order elastic strain tensor.

The thermal strain *ε^th^* is given by [39]:

*ε^th^* = *α*·*∆T* (6)

where *α* is the thermal expansion coefficient and *ΔT* is the temperature change in a certain time duration.

### 3.3. Heat Source Model and Material Properties

In temperature field analysis, the heat flux density load with uniform power density has been developed to model the heat input of the laser. The heat flux is applied to the active element of the powder via an ANSYS APDL subroutine. The heat source was considered a constant and uniformly distributed body heat flux, defined as:

(7) $$Q=\frac{\alpha P}{\pi r^{2}t}$$

where *α* is the laser absorption coefficient, set as 0.3 according to the experiments, *P* is the power of the continuous laser, *r* is the radius of the laser beam, and *t* is the layer thickness.

The thermophysical properties and mechanical properties were temperature-dependent and identified in [35,40], as shown in Table 6.

## 4. Results and Discussion

### 4.1. Temperature Evaluation

**Figure 7:**
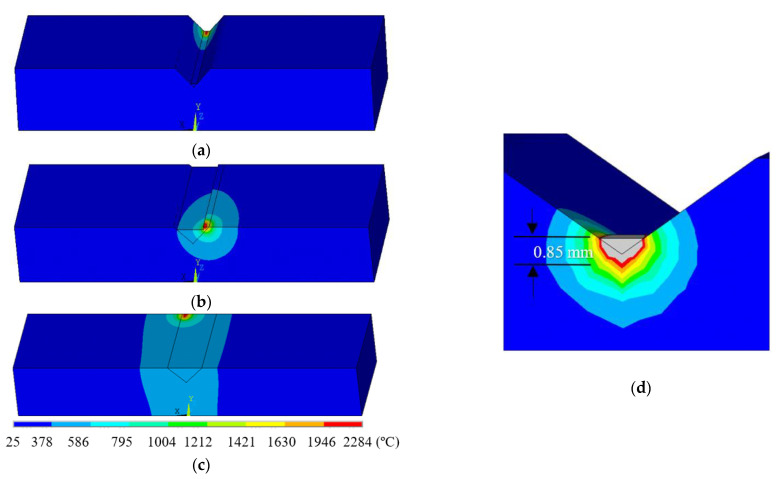
Temperature distribution at the end of the nth track, n is 1 (**a**), 16 (**b**), and 21 (**c**). The zoom-in temperature contours of the first track are shown in (**d**).

Figure 7 presents the simulated temperature distribution at each deposition in the triangular defect case, where the laser is applied to the end of the 1st track in Figure 7a, followed by the 16th and 21st tracks in Figure 7b,c, respectively. The deposition of the first track generates a temperature of 1854 °C, and the substrate remains at a transient room temperature. Measured from the zoom-in temperature contours, the depth of the area where the temperature was higher than the melting point was 0.85 mm, as shown in Figure 7d (the zone encompassed by the grey color depicted in the melt pool). It can be observed that the melt depth was deep enough to enable fully metallurgical bonding between the first deposited layer and the base substrate. The deposition of the 16th and 21st tracks experienced temperatures of 2029 °C and 2284 °C, respectively, but higher temperatures were observed in the substrate near the heat-affected zone compared to the first track due to the thermal accumulation.

**Figure 8:**
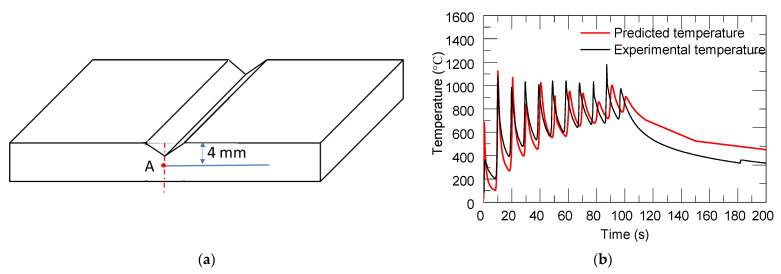
(**a**) The locations of each measurement, Point A (TC1); (**b**) the simulated and measured temperature history.

Figure 8b shows the simulated temperature history during the deposition process at the selected position, Point A, as depicted in Figure 8a. Point A is located at the side surface of the substrate. Point A corresponds to experimental measurements at the thermocouple location TC1 of the substrate. The temperature evolution obtained from numerical simulations and experimental measurements during the real manufacturing process are compared in Figure 8b. The red line and black lines indicate predicted temperature and experimental measurement data, respectively. It was observed that both simulation and experimental observations followed the same trends. The agreement is notable. The heating stage corresponded to the deposition of the sequence of 21 deposited layers until completing the build, including 11 temperature peaks. After the deposition process was completed, the accumulated heat gradually dissipated, and the substrate gradually cooled down.

### 4.2. Distortion and Stress Evaluation

**Figure 9:**
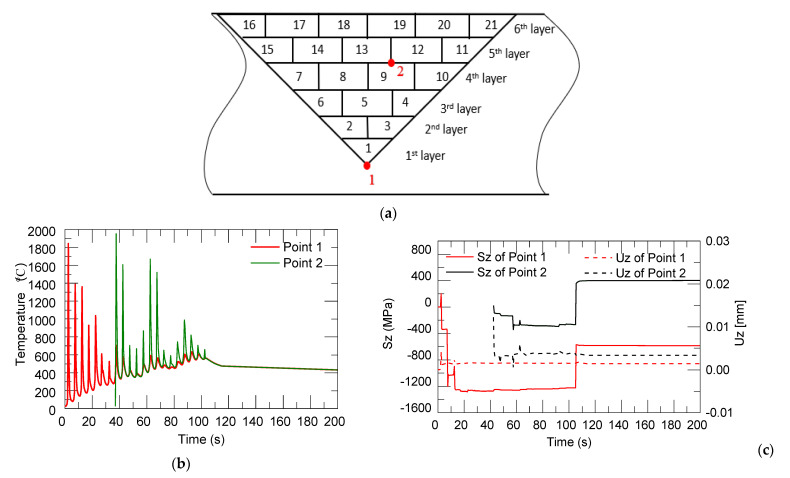
Location of points 1 and 2 (**a**). Curve graph of cycles in temperature (**b**), thermal stress and distortion (**c**) on the first and ninth track.

Figure 9 shows the thermal stress and distortion evaluation in the longitudinal direction (along the laser moving direction) of two selected points, 1 and 2, which are located at the mid-points of the first and ninth deposition tracks (shown in Figure 9a; point 1 is in the substrate). Both numerical curves show several peaks due to the regular movement of the laser heat source to deposit the different layers. As the laser heat source approaches the selected point, the temperature and thermal stress at this point rise rapidly to the highest point. When the heat source is far away, the temperature and the thermal stress will gradually decrease until it is stable. After the depositing started, the temperature and thermal stress of point 1 were relatively high, with a temperature of 1854 °C. The thermal stress was in the tensile state, with a value of 199 MPa due to the material expansion by laser irritation. The deformation was relatively high because it was located at the bottom of the V-shaped groove. As the laser heat source moved, the temperature and thermal stress kept changing. With the movement of the heat source to the upper layers and far away from point 1, the temperature and thermal stress also showed a decreasing trend. When the deposition was completed, the accumulated heat gradually dissipated, and the part gradually cooled down. Material shrinkage was shown in the compressive state due to the cooling and shrinking of these deposited layers. This explains the longitudinal stress distributions (Sz) (along the laser moving direction) for different process times, shown in Figure 9c.

**Figure 10:**
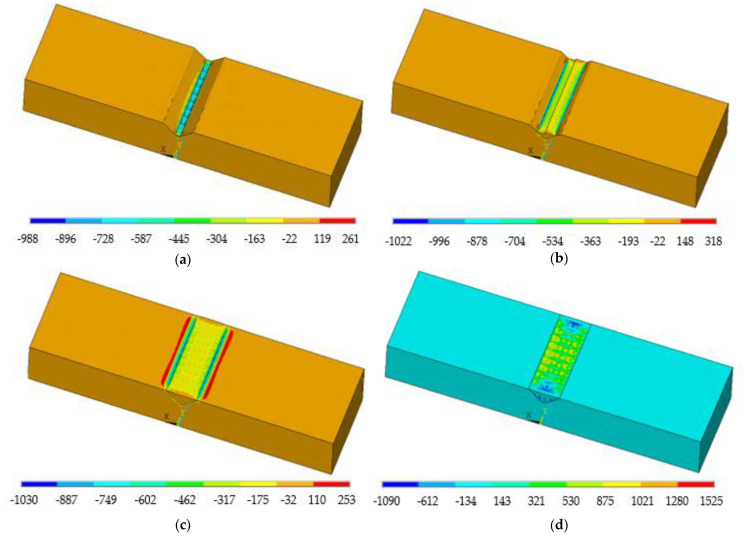
Longitudinal stress distribution at the end of the 1st (**a**), 4th (**b**), and final (**c**) tracks, and after cooling to room temperature (**d**).

Figure 10a shows the variations in the longitudinal stress after the first track. The thermal expansion of the melt pool led to large compressions in the material around this zone. Next, as the melt pool moved to the 8th track in the fourth layer (Figure 10b), the maximum compressive stresses were transferred to the heat affected zone (HAZ) below the melt pool. After the deposition (Figure 10c), large longitudinal tensile stresses of 1030 MPa were induced at the interface with the substrate. The continuous cooling to room temperature resulted in very high tensile stress values of 1525 MPa at the top surface of the substrate.

The sum displacement and von Mises stress distribution after the deposition was completed and after cooling to room temperature in the triangular case are illustrated in Figure 12a,b. The maximum displacement was formed at the interface region of the building part, with a value of 0.08 mm. Near the bottom surface of the deposited layer, the highest residual stress was observed, with a value of 797 MPa. Residual stress could also introduce some deformation problems to the damaged plate. In the current study, the average equivalent stresses showed low values compared with the yield stress of Ti6Al4V (σ_y_ = 954 MPa). As a result, no cracks were observed in deposited layers and bonding areas (Figure 4). Hence, this laser repair task can be considered successful. The sum displacement and von Mises stress distribution after the deposition was completed until cooling to room temperature in the rectangular case are illustrated in Figure 12c,d. It was observed to have the same trend as the first case in respect to the sum displacement and von Mises stress distribution, except for a slightly higher value of 0.122 mm in the displacement vector. The average equivalent stresses showed that near the bottom of the deposition some values exceeded the yield stress, which may induce cracks or delamination there.

**Figure 11:**
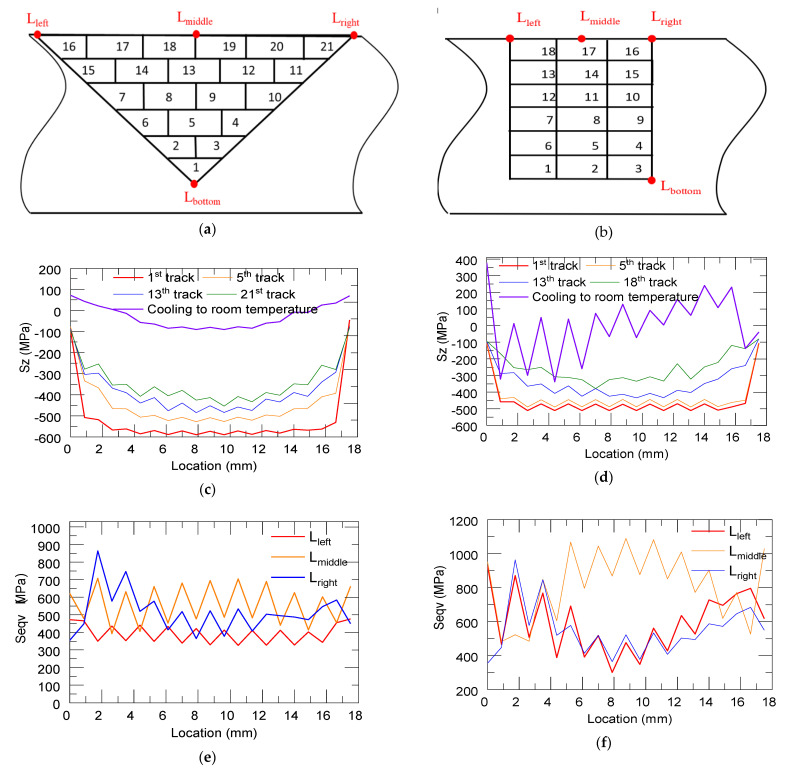
Schematic showing the definition of L_left_, L_right_, L_middle_, and L_bottom_ for the triangular (**a**) and rectangular (**b**) case; longitudinal residual stress distribution along the interface line after the deposition of 1st, 5th, 13th, and last layers in the triangular (**c**) and rectangular (**d**) repair cases; Von Mises stress distributions along three lines in the triangular (**e**) and rectangular (**f**) repair cases.

**Figure 12:**
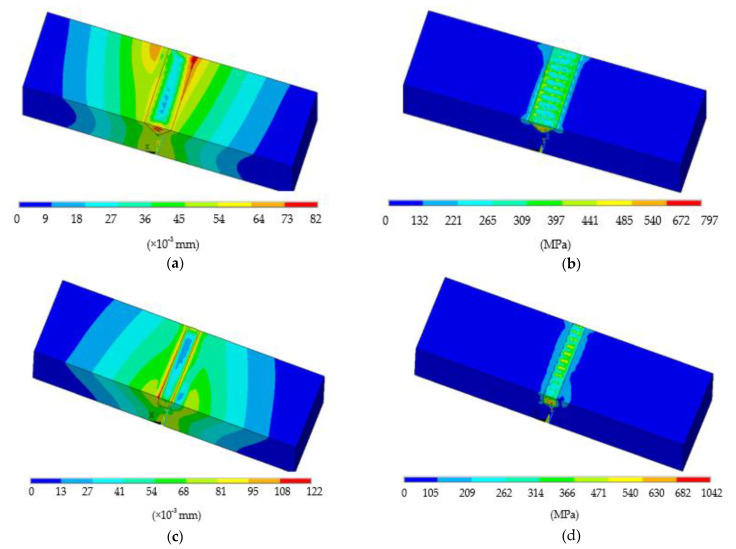
(**a**) Displacement vector sum, Usum; (**b**) Von Mises stress, Seqv, after deposition and cooling to room temperature in triangular (**c**) and rectangular (**d**) cases.

Figure 11c,d show the longitudinal stress distribution (Sz) along the interface line between the substrate and the deposition (L_bottom_ refers to the interface line between the deposit and the substrate on the bottom surface) after the deposition time of 1st, 5th, 13th, and last layers and cooling to room temperature in the triangular and rectangular repair cases, respectively. The locations of different lines are depicted in Figure 11a,b, in which the left and right lines refer to the interface lines between the deposit and substrate on the top surface, L_left_ and L_right_. The middle line is represented by L_middle_ of the deposit on the top surface. The magnitude of the thermal stress near the center decreased with the progress of the deposition process due to repetitive heating and cooling. At the end of the deposition process, the top free end of the deposit cooled down to room temperature at the very end. Because of this, tensile stress accumulated near the top free end. This resulted in the accumulation of large compressive stress near the substrate–deposit interface. After the deposition was completing, due to the cooling and shrinking of these deposited layers, the distortion of the substrate sharply decreased. When cooled to room temperature, the magnitude of the compressive stress and the stress quickly transitioned into tensile stress. Hence, almost all the residual distortion developed during the initial part of the cooling phase. This can explain the trend shown in Figure 11c. The same trend was observed in the two cases and there were some differences when cooling to room temperature, which may be due to a vertical sidewall on the bottom region. The longitudinal stress after the deposition was completed and after cooling to room temperature in the rectangular repair case was slightly higher than that in the triangular case. This situation may be worsened when the defect is deep and when the open area is narrow. Figure 11e,f show the von Mises stress distributions along three lines after cooling to room temperature in the triangular and rectangular repair cases, respectively. When referring to L_left_ and L_right_, we observed the same trend and magnitude level with each case in respect to the von Mises stress distribution. As for the middle line, the von Mises stress values of the rectangular case (600–700 MPa) were higher than those of the triangular case (800–1000 MPa).

## 5. Conclusions

In this study, we have presented a damage reconstruction method for V-shaped groove repairs using the directed energy deposition (DED) process. The damaged parts had triangular or rectangular defects in cross-section. In this work, we investigated the feasibility of repairing two V-shaped defects with both experiments and modeling. The main conclusions are indicated below.

Ti6Al4V powder particles were deposited on the damaged region using the DED technique, following the repair toolpath. The microstructure analysis and tensile testing confirmed solid bonding along the interface for the triangular defect repair case. The tensile test showed an average ultimate tensile strength of 1037 MPa for the repaired parts, and samples fractured at the region of the deposits, not at the interface. However, for repairing the sample with rectangular damage, the filler material did not bond well with the substrate, causing a large number of pores.

3D finite element models based on sequentially coupled thermo-mechanical field analysis were developed to simulate the corresponding repair deposition processes. Finally, the average equivalent stresses and deformation in the triangular repair case showed low values at the intersection between the base plate and the deposited tracks than those in the rectangular repair case. The high equivalent stresses near the bottom deposition may induce cracks or delamination in the rectangular repair case. The predicted deformation and stress results will guide the evaluation of the quality of repaired parts based on repair slot geometry.

## Figures and Tables

**Table 1:** Chemical composition of the target materials (wt.%).

Material	C	V	Fe	H	Al	O	Ti
Ti6Al4V	0.08	4.5	0.25	0.025	6.76	0.2	Balance

**Table 2:** Processing parameters for the repair experiment.

Experiment Parameters	Values
Power	650 W
Powder flow rate	2.8 g/min
Scan speed	210 mm/min
Laser diameter	1 mm
Layer thickness	0.5 mm

**Table 3:** Yield strength (YS) and ultimate tensile strength (UTS) of the repaired parts.

Sample #	YS (MPa)	UTS (MPa)
1	862.08	1046.32
2	1011.82	1069.93
3	996.71	1058.80
4	905.13	1010.41
5	984.27	1029.54
6	962.05	1034.43
7	934.77	1028.68
8	955.25	1006.12
9	978.30	1050.29
Average	954.49	1037.17
S.D.	47	21

**Table 4:** Input parameters for the numerical simulation.

Experimental Parameters	Values
Power	650 W
Laser absorptivity	0.3
Effective scan speed	210 mm/min
Laser diameter	1 mm
Time step	0.1 s

**Table 5:** The convection heat transfer coefficient used in the simulation.

**Temperature (°C)**	25	200	400	600	800	1000	1500	2000
***h* (W/(m^2^·K))**	12	24	40	72	80	100	100	100

**Table 6:** Thermo-mechanical properties of the Ti6Al4V.

Temperature (°C)	25	100	200	300	400	600	800	1200	1300	1600
Density, (kg/m^3^)	4420	4406	4395	4381	4366	4336	4309	4252	4240	3920
Thermal conductivity, (W/(mK))	7	7.45	8.75	10.15	11.35	4.2	17.8	23	24	50
Specific heat, (J/(kgK))	500	502	505	510	513	518	522	530	530	530
Thermal expansion coefficient, (10^−6^/K)	10	10	10	10	10	10	10	11	11	11
Poisson’s ratio	0.33	0.33	0.33	0.33	0.33	0.33	0.33	0.33	0.33	0.33
Elastic modulus, (GPa)	125	120	115	105	93	40	25	15	12	8
Yield strength, (MPa)	850	720	680	630	590	490	40	5	1	0.1

## Data Availability

The data presented in this study are available on request from the corresponding author.
